# The associations between anxiety/depression and plasma chromogranin A among healthy workers: Results from EHOP study

**DOI:** 10.1002/1348-9585.12113

**Published:** 2020-02-07

**Authors:** Ying Li, Yao Song, Weimin Dang, Lijun Guo, Weixian Xu

**Affiliations:** ^1^ Department of Cardiology and Institute of Vascular Medicine Peking University Third Hospital Key Laboratory of Cardiovascular Molecular Biology and Regulatory Peptides, Ministry of Health Key Laboratory of Molecular Cardiovascular Science, Ministry of Education Beijing Key Laboratory of Cardiovascular Receptors Research Beijing China; ^2^ Peking University Sixth Hospital Peking University Institute of Mental Health NHC Key Laboratory of Mental Health (Peking University) National Clinical Research Center for Mental Disorders (Peking University Sixth Hospital) Beijing China

**Keywords:** anxiety, catestatin, chromogranin A, depression

## Abstract

**Objectives:**

Chromogranin A (CgA) is regarded as an indicator of sympathetic tone and adrenomedullary system activity. Catestatin is one of CgA‐derived fragments through proteolytic processing. Many studies have confirmed the correlation between anxiety/depression and the salivary CgA level. The study was to investigate the associations between anxiety/depression and plasma CgA/catestatin levels in healthy workers without cardiovascular disease.

**Methods:**

The study included 263 healthy workers (175 men and 88 women). The symptoms of anxiety and depression were measured with the Hospital Anxiety and Depression Scale (HADS). Plasma CgA and catestatin levels were measured by ELISA kits.

**Results:**

In bivariate correlation analysis, anxiety and depression were positively associated with plasma CgA level, respectively (r = 0.298, *P* < .001; r = 0.304, *P* < .001), but not significantly associated with plasma catestatin level.

The anxiety group had significantly higher plasma CgA level than that in the no‐anxiety group (median 158.60 vs 70.90, *P* < .001). The similar results were found for depression scales. The depression group had significantly higher plasma CgA level (median 145.60 vs 82.40, *P* < .001).

In the multiple linear regression model, after adjusting for age, gender, and BMI, anxiety was positively correlated with plasma CgA level (β = 0.359, *P* < .001), while anxiety was negatively correlated with plasma catestatin level (β = −0.128, *P* = .044), depression was also positively correlated with plasma CgA level (β = 0.343, *P* < .001).

**Conclusions:**

Plasma CgA was associated with anxiety and depression in healthy workers. It can be considered as the blood indicator for the evaluation of anxiety and depression.

## 1 INTRODUCTION

Chromogranin A (CgA) is a member of the granins, a family of acidic proteins. It is expressed by several normal or neoplastic cells of the diffuse endocrine and neuroendocrine systems or by some cancer cells. CgA is co‐stored and co‐released with catecholamines from storage granules in the adrenal medulla, or with the parathyroid hormone in response to hypocalcemia in the parathyroid gland.^1^ Previous studies have confirmed that CgA correlated with norepinephrine release rate when they compared CgA concentration and neurotransmitters concentrations.^2^, ^3^ As CgA is much more stable than catecholamines in the circulatory system, its plasma level reflects the sympathetic tone and adrenomedullary system activity. CgA can be cleaved into several bioactive fragments, such as vasostatin, pancreastatin, catestatin, and serpinins, which exert abroad spectrum of regulatory activities by influencing the endocrine, the cardiovascular, and the immune systems and by affecting the glucose or calcium homeostasis.^4^ Previous studies reported that high CgA plasma level was strictly associated with mortality risk after acute coronary syndrome as well as heart failure.^5^, ^6^

Catestatin is one of CgA‐derived fragments through proteolytic processing, which is an endogenous multifunctional neuroendocrinepeptide. Catestatin can induce cardiovascular responses at local as well as at systemic levels. Catestatin inhibits catecholamine release from both chromaffin cells and noradrenergic neurons in a reversible and noncompetitive fashion.^7^ It induces vasorelaxant by means of the induction of histamine release from mast cells.^8^ Catestatin also induces monocyte chemotaxis^9^ and exhibits pronounced angiogenic and vasculogenic activities.^10^ In addition, catestatin directly inhibits growth of fungi, yeast, and bacteria.^11^ A number of studies have shown that catestatin is closely related to many cardiovascular diseases, such as hypertension^12^ and acute coronary syndrome.^13^ It is correlated with left ventricular ejection fraction (LVEF)^14^ and malignant arrhythmia in acute AMI stage,^15^ as well as with left ventricular remodeling 3 months later^14^ and the severity of heart failure.^16^

Anxiety and depression are a highly prevalent condition, they are also a growing global problem. Over the past decades, a cumulative number of studies have shown that anxiety and depression can affect the incidence and prognosis of cardiovascular diseases such as hypertension, coronary heart disease, and heart failure.^17^, ^18^, ^19^, ^20^ It should be noted that a scientific advisory statement from the AHA published in 2014 critically reviewed the evidence for depression as a risk factor for morbidity and mortality after an ACS.^21^ Numerous potential mechanisms have been postulated for the associations between anxiety or depression and cardiovascular diseases, neuroendocrine dysfunction is one of the main candidate mechanisms. Acute and chronic stress exposure can lead to the changes of neurochemical function, such as disruptions in the synthesis or activity of norepinephrine, dopamine, or serotonin,^22^ they can also lead to dysfunction of hypothalamic‐pituitary‐adrenocortical system,^23^ which, in turn, may influence cardiovascular risk.

CgA is regarded as an indicator of sympathetic tone and adrenomedullary system activity. In the study of the associations between anxiety/depression and CgA, most studies have confirmed the correlation between anxiety/depression and the level of CgA. Matsumoto T found that salivary CgA level significantly and positively correlated with total mood disturbance during intramenstrual cycle in women.^24^ In a 2011 study, there were significant differences in levels of awakening salivary CgA between depressed patients and non‐depressed patients in healthy individuals.^25^ However, previous studies mostly focused on salivary CgA level, and few focused on plasma/serum CgA level. Therefore, the associations between plasma CgA level and anxiety/depression are unknown. Many studies have shown that plasma CgA and catestatin levels play important roles in cardiovascular diseases, so we speculated that anxiety/depression might play roles in cardiovascular diseases by changing plasma CgA and catestatin levels. Working population is an important part of social productivity, their health status directly affect the development of social productivity. At the same time, they may face more mental problems, so we need to pay enough attention to their health status. The aim of our study was to investigate the associations between anxiety/depression and plasma CgA/catestatin levels in healthy workers without cardiovascular disease.

## 2 METHODS

### 2.1 Subjects

The Emotion and Health among Occupational Population (EHOP) study was a registry study to explore the associations between emotional factors and physical health among Chinese occupational population. There were 300 occupational individuals underwent physical checkups at the Peking University Third Hospital in August 2016. Participants who had a history of chronic disease, such as cardiovascular disease, cerebrovascular disease, hypertension, hyperlipidemia, diabetes mellitus, or cerebrovascular disease were excluded. This study was performed in accordance with the Declaration of Helsinki and was approved by the ethnic board of the Peking University Third Hospital. All the subjects provided their written informed consent.

### 2.2 Questionnaire of anxiety and depression

The symptoms of anxiety and depression were measured with the Hospital Anxiety and Depression Scale (HADS), a self‐report questionnaire with a 7‐item anxiety (HADS‐A) and 7‐item depression (HADS‐D) subscale.^26^ Items are scored on a 4‐point Likert scale ranging from 0 to 3, with a score range of 0‐21 for both subscales. A cut‐off score ≥8 was used to indicate probable clinical levels of anxiety and depression, respectively.^27^ The HADS is a valid and reliable instrument that has been used across the world and is not prone to confounding by symptoms of somatic disease.^28^

### 2.3 Demographic characteristics

The demographic characteristics, such as age, gender, marriage, education, body mass index (BMI), were collected from medical records.

### 2.4 Assays for blood sample

Fasting blood samples were collected from 8:00 to 10:00 on the day of physical examination with one‐time venous blood sampling needle. Venipuncture at the median cubital vein was performed by the same trained nurse with the subject seated in a chair. Blood samples were taken into chilled EDTA vacutainers containing 2500 IU/mL aprotinin, then immediately centrifuged at 3000 rpm for 10 minutes at 4°C, then finally stored at −80°C till analysis. Plasma CgA levels were determined using standard enzyme‐linked immunosorbent assay (ELISA) kits (MyBioSource). Plasma catestatin levels were measured by the catestatin ELISA kits (Phoenix Pharmaceuticals) according to the manufacturer's instructions.

### 2.5 Statistical analysis

Continuous variables which were normally distributed were given as mean ± SD, continuous variables were not normally distributed presented as medians (25%, 75%), and categorical variables were defined as percentage. Bivariate correlation analysis (Pearson method or Spearman method) was used for the relationship of two continuous variables. The Mann‐Whitney *U* test was used to compare plasma CgA/catestatin levels between anxiety group and no‐anxiety group, as well as the differences between depression group and no‐depression group. The multiple linear regression was used to evaluate the associations between anxiety/depression and CgA/catestatin. Six different regression models were formed: model 1 referred to the associations between anxiety and CgA; model 2 referred to the associations between anxiety and catestatin; model 3 referred to the associations between depression and CgA; model 4 referred to the associations between depression and catestatin; model 5 referred to the associations between anxiety, depression, and CgA; and model 6 referred to the associations between anxiety, depression, and catestatin; all the models were adjusted the confounders such as age, gender, and BMI. All tests of significance were two‐tailed. Statistical significance was defined as *P* ＜ .05. The statistical analysis was performed by SPSS statistical software (SPSS 20.0 for Windows; SPSS Inc).

## 3 RESULTS

From the 300 eligible people, 32 subjects were excluded because of chronic diseases history, and there were five incomplete questionnaires. So 263 subjects were included in the final analysis including 175 men (66.5%) and 88 women (33.5%). The average age was 37.8 ± 10.7 years old, with an average BMI of 23.9 ± 3.3 kg/m^2^. In terms of education, 82 individuals (31.1%) had less than 12 years of education, 83 individuals (31.6%) had a bachelor's degree, and 98 individuals (37.3%) had a master's degree or above. In terms of the occupational backgrounds, 134 individuals (51.0%) were white collars, 26 individuals (9.9%) were civil servants, 41 individuals (15.6%) were blue collars, and 62 individuals (23.6%) were other occupations such as businessmen and freelancers.

The Cronbach's alpha coefficient of HADS‐A scale was 0.89, and that of HADS‐D scale was 0.80. Among the subjects, 48.3% (127) of participants scored ≥8 on the HADS‐A scale and 41.4% (109) scored ≥8 on the HADS‐D scale. The average score of the HADS‐A scale was 6.9 ± 4.8, and that of the HADS‐A scale was 6.3 ± 3.9.

The range of plasma CgA level was 0.10 ng/mL to 744.50 ng/mL, and its median was 109.30 (57.70, 199.70) ng/mL; the range of plasma catestatin level was 0.37 ng/mL to 4.62 ng/mL, and its median was 1.41 (1.21, 1.65) ng/mL. In bivariate correlation analysis, anxiety and depression were positively associated with plasma CgA level, respectively (r = 0.298, *P* < .001; r = 0.304, *P* < .001), but not significantly associated with plasma catestatin level. In the meanwhile, anxiety was positively associated with depression (r = 0.841, *P* < .001).

As shown in Table 1, the participants were divided into two groups (anxiety/no‐anxiety groups or depression/no‐depression groups). The anxiety group had significantly higher plasma CgA level than that in the no‐anxiety group (median 158.60 vs 70.90, *P* < .001). The similar results were found for depression scales. The depression group had significantly higher plasma CgA level (median 145.60 vs 82.40, *P* < .001).

**Table 1:** The differences of plasma CgA and catestatin levels between groups^a^

Variables	No‐anxiety group (n = 136)	Anxiety group (n = 127)	*P* value	No‐depression group (n = 154)	Depression group (n = 109)	*P* value
CgA (ng/mL)	70.90 (48.25, 149.80)	158.60 (80.80, 236.30)	<.001	82.40 (51.03, 177.15)	145.60 (76.55, 233.20)	<.001
Catestatin (ng/mL)	1.44 (1.24, 1.78)	1.36 (1.19, 1.62)	.090	1.42 (1.22, 1.65)	1.38 (1.17, 1.66)	0.532

a The CgA or catestatin levels were presented as medians (25%, 75%).

In the multiple linear regression model, after adjusting for age, gender, and BMI, anxiety was positively correlated with plasma CgA level (β = 0.359, *P* < .001), while anxiety was negatively correlated with plasma catestatin level (β = −0.128, *P* = .044), depression was also positively correlated with plasma CgA level (β = 0.343, *P* < .001). When both anxiety and depression were included in the multiple linear regression model, anxiety was also positively correlated with plasma CgA level (β = 0.251, *P* = .021) and negatively correlated with plasma catestatin level (β = −0.248, *P* = .031), while depression was not independently correlated with plasma CgA and catestatin (Table 2).

**Table 2:** The associations between anxiety/depression and plasma CgA/catestatin levels in the multiple linear regression model

Variables	B	SE	β	t	*P*
Model 1: the associations between anxiety and CgA					
Constant	183.586	58.715		3.127	.002
Age	0.917	0.556	0.098	1.650	.100
Gender	−7.349	13.766	−0.035	−0.534	.594
BMI	−5.109	1.966	−0.169	−2.599	.010
Anxiety	7.468	1.238	0.359	6.030	<.001
Model 2: the associations between anxiety and catestatin					
Constant	1.672	0.342		4.883	<.001
Age	0.003	0.003	0.050	0.792	.429
Gender	0.131	0.080	0.112	1.633	.104
BMI	−0.014	0.011	−0.083	−1.210	.227
Anxiety	−0.015	0.007	−0.128	−2.026	.044
Model 3: the associations between depression and CgA					
Constant	180.331	59.400		3.036	.003
Age	1.066	0.561	0.114	1.899	.059
Gender	−4.959	13.951	−0.023	−0.355	.723
BMI	−5.469	1.984	−0.181	−2.756	.006
Depression	8.688	1.539	0.343	5.646	<.001
Model 4: the associations between depression and catestatin					
Constant	1.619	0.346		4.682	<.001
Age	0.002	0.003	0.048	0.756	.451
Gender	0.138	0.081	0.118	1.700	.090
BMI	−0.014	0.012	−0.083	−1.189	.235
Depression	−0.009	0.009	−0.064	−0.996	.320
Model 5: the associations between anxiety, depression, and CgA					
Constant	177.498	58.886		3.014	.003
Age	0.979	0.558	0.105	1.755	.081
Gender	−5.627	13.830	−0.027	−0.407	.684
BMI	−5.266	1.968	−0.174	−2.676	.008
Anxiety	5.225	2.250	0.251	2.322	.021
Depression	3.310	2.773	0.131	1.193	.234
Model 6: the associations between anxiety, depression, and catestatin					
Constant	1.635	0.343		4.761	<.001
Age	0.003	0.003	0.057	0.906	.366
Gender	0.142	0.081	0.121	1.758	.080
BMI	−0.015	0.011	−0.089	−1.293	.197
Anxiety	−0.028	0.013	−0.248	−2.165	.031
Depression	0.020	0.016	0.146	1.256	.210

Note

The six models adjusted for age, gender, and BMI.

## 4 DISCUSSION

In this study, the plasma CgA level was higher in patients with anxiety or depression. Anxiety/depression was independently associated with plasma CgA level. While the catestatin level was negatively associated with anxiety.

To the best of our knowledge, the present study is the first study to investigate the associations between anxiety/depression and plasma CgA/catestatin among healthy individuals. Our results were in accordance with the previous studies. Serfozo G found that the increased plasma CgA were associated with low social support and severe depressive symptoms in 23 patients with stable coronary heart disease.^29^ In addition, some Japanese scholars have conducted a large number of studies on the relationship between salivary CgA level and anxiety or depression. Most of these studies have confirmed a correlation between salivary CgA level and anxiety or depression, while a few did not. Matsumoto T investigated whether salivary CgA changed during the menstrual cycle in women with different degrees of premenstrual psychoemotional symptoms, used the Profile of Mood State to assess current mood states, they found that salivary CgA level significantly and positively correlated with total mood disturbance and four emotional subscales: tension‐anxiety, depression‐dejection, anger‐hostility, and confusion in the late‐luteal phase.^24^ Similarly, in a study of healthy people, Den R found significant differences in levels of awakening salivary CgA between depressed patients and non‐depressed patients.^25^ However, Kaneko S found that the level of saliva CgA in patients with atopic dermatitis was unrelated to the State‐Trait Anxiety Index scores.^30^ Plasma and salivary CgA had different routes of secretion. Saruta demonstrated that salivary CgA was produced in the serous and ductal cells of the human submandibular gland.^31^ Meanwhile, plasma CgA was secreted with catecholamines from the adrenal medulla and sympathetic nerve endings.^32^ There are few studies on the correlation between plasma and salivary CgA level, and the results are inconsistent. Dag E confirmed their correlation in epileptic cases,^33^ while another study found no correlation between them.^34^ Therefore, the correlation between plasma and salivary CgA level needs to be further explored. In our study, plasma CgA level was significantly correlated with anxiety and depression. It supports that plasma CgA can be considered as an objective index for the evaluation of anxiety and depression.

Previous studies have shown that neuroendocrine dysfunction is one of the mechanisms underlying the relationship between anxiety or depression and cardiovascular disease. The level of plasma CgA reflects the sympathetic tone and adrenomedullary system activity. In our study, we found that plasma CgA levels have increased significantly in healthy individuals with anxiety or depression. Therefore, we believe that the activation of sympathochromaffin/CgA axis may play an important role in the development of cardiovascular disease in anxious and depressed individuals. In the future, we may further explore whether plasma CgA levels can be used to determine the future risk of cardiovascular disease in these populations.

Catestatin is an endogenous multifunctional neuroendocrinepeptide. It has been shown to play a role in a variety of cardiovascular diseases and is considered to have cardiovascular protective effects. In our study, decreased plasma catestatin levels in anxious patients are consistent with their susceptibility to cardiovascular disease. However, catestatin is one of CgA‐derived fragments through proteolytic processing, what we found was that plasma CgA levels were increased, but plasma catestatin levels were lower in anxious patients. There is no clear explanation for this phenomenon at present. Recently, a study^35^ by Ottesen may provide some answers. In their animal studies, CgA was hyperglycosylated in the failing myocardium, in clinical studies, they found that acute heart failure patients with low CgA‐to‐catestatin conversion had a worse outcome compared with the patients with higher CgA‐to‐catestatin conversion. They thought that myocardial glycosylation was found to be increased in the failing myocardium, it represented a mechanism for posttranslational protein modification, processing of glycosylated proteins could be impaired by sugar groups blocking the binding of proteases to cleavage sites. As CgA processing takes place at several dibasic cleavage sites along the full‐length molecule, extensive CgA glycosylation could influence CgA processing. Additional mechanism for catestatin removal may be degradation pathways of CgA to fragments other than catestatin or factors that independently affect the breakdown or stability of catestatin, which both will have to be assessed in additional studies.

In our study, we found that anxiety was positively correlated with depression. Regardless of the presence or absence of depression, anxiety was correlated with plasma CgA and catestatin levels, while depression was no longer correlated with plasma CgA level after adjusting for anxiety. Therefore, the association between depression and plasma CgA level may be caused by anxiety.

In addition, Pereda D established three kinds of mice with CgA gene knocked out, CgB gene knocked out and double genes knocked out, they found that the lack of CgB, overexpressing CgA, produced a depressive‐like and aggressive phenotype.^36^ It unveiled the existence of direct implications of Cgs in the control of behavior and mood beyond what was known. Based on the results of this experiment, combined with the findings of our study and previous studies, we try to speculate that another possibility is that the difference of CgA levels in the population are predisposing factors: on the one hand, the elevation of initial level of CgA leads to mood disorders, and on the other hand it increases the incidence of cardiovascular disease, both of which are comorbidities. But this is only a direction that can be considered and requires a lot of research to clarify.

Several limitations of this study need to be addressed. First, as our study was cross‐sectional, we could not draw a cause‐effect conclusion. Second, this was also only one center study. Third, in our study, the prevalence of anxiety and depression seemed to be very high. On the one hand, the prevalence of anxiety and depression in China is actually very high. As shown in a study of Chinese college students, over 45% of female students and around 40% of male students reported experiencing anxiety above the normal threshold in the freshman year, meanwhile, around 32% of all students above the normal levels of depression in the freshman year.^37^ On the other hand, we might overestimate the incidence of anxiety and depression by ≥8 points as the assessment of anxiety and depression. When HADS is used to evaluate anxiety and depression, 8‐10 points are judged as suspicious existence, and 11‐21 points are determined as positive existence. Furthermore, people with anxiety or depression are more likely to have physical examination. Our study was to investigate the associations between anxiety/depression and plasma CgA/catestatin levels. If there was overestimation of anxiety or depression, the overestimation might lead to reduction of the differences between groups. Even under this conditions, the differences in CgA or catestatin between groups were significant. Fourth, needle insertion may cause pain, anxiety, and stress during blood sample collection, so we need to consider whether this reaction will affect the levels of CgA and catestatin. There is no direct research on this aspect, and most of the relevant research focus on children. Although it might affect the levels of CgA and catestatin, all individuals were collected blood samples under the same conditions, so the differences between them are not affected. Fifth, only age, gender, and BMI were adjusted in the multiple linear regression model for the associations of anxiety/depression with CgA/catestatin. It may not be a complete adjustment, there may be other potential confounders, but our study subjects are all healthy workers without chronic diseases, and there are no factors clearly related to the levels of CgA/catestatin. Therefore, we only adjusted the conventional factors.

In summary, we found that anxiety/depression was positively associated with plasma CgA, while anxiety was negatively associated with catestatin levels in healthy workers. The plasma CgA could be considered as the blood indicator for the evaluation of anxiety and depression. Furthermore, we thought that the activation of sympathochromaffin/CgA axis may play an important role in the development of cardiovascular disease in anxious and depressed people. Further research is needed to verify it. But there is no doubt that we need to take more measures to improve the mental problems of the working population, and we may consider plasma CgA as the evaluation of the intervention effect.

## DISCLOSURE

*Approval of the research protocol:* this study was performed in accordance with the Declaration of Helsinki and was approved by the ethnic board of the Peking University Third Hospital. *Informed consent:* all persons gave their informed consent prior to their inclusion in the study. *Registry and the registration no. of the study:* N/A. *Animal studies:* N/A. *Conflict of interest:* Authors declare no conflict of interests for this article.

## AUTHOR CONTRIBUTIONS

WXX and WMD conceived the ideas; YL and Y.S collected the data; YL and WXX analyzed the data; and YL, WXX, and LJG led the writing.
